# Biochemical bone biomarkers in plasma cell dyscrasias

**DOI:** 10.1111/bjh.70467

**Published:** 2026-04-08

**Authors:** Guido Nador, Rohit Vijjhalwar, Muhammad Kassim Javaid, Claire M. Edwards, Karthik Ramasamy

**Affiliations:** ^1^ Oxford University Hospitals NHS Foundation Trust Oxford UK; ^2^ University Hospital Southampton NHS Foundation Trust Southampton UK; ^3^ Nuffield Department of Orthopaedics, Rheumatology and Musculoskeletal Sciences University of Oxford Oxford UK; ^4^ Nuffield Department of Surgical Science University of Oxford Oxford UK; ^5^ Oxford Translational Myeloma Centre NDORMS, University of Oxford Oxford UK

**Keywords:** bone morbidity, bone turnover markers, MGUS, myeloma

## Abstract

Multiple myeloma (MM) is a haematological malignancy characterised by clonal plasma cell accumulation in the bone marrow, frequently resulting in myeloma bone disease. Disruption of bone homeostasis, driven by increased osteoclastic resorption and suppressed osteoblastic formation, leads to osteolytic lesions and skeletal‐related events, contributing substantially to morbidity and mortality. Bone turnover markers (BTMs) provide a non‐invasive means of assessing bone metabolism, with potential applications in disease monitoring, prognostication, risk stratification from monoclonal gammopathy of undetermined significance (MGUS) to MM and evaluation of response to myeloma and osteoporosis therapies. This review examines the clinical relevance of BTMs in plasma cell dyscrasias, including MM, MGUS and smouldering MM. Bone formation markers, such as bone‐specific alkaline phosphatase and procollagen type I N‐terminal propeptide, reflect osteoblastic activity, while resorption markers, including carboxy‐terminal collagen crosslinks and deoxypyridinoline, indicate osteoclastic activity. Although several BTMs correlate with disease burden and progression, their routine clinical use is limited by biological variability, renal impairment and lack of assay standardisation. As myeloma therapies continue to evolve, further research is needed to define the role of BTMs alongside imaging and clinical risk models to optimise bone disease management.

## Graphical abstract

Visual abstract depicting that bone turnover markers reflect dynamic alterations in bone remodelling across the spectrum of plasma cell dyscrasias but remain limited by assay variability, biological confounding and incomplete integration with imaging and risk stratification.

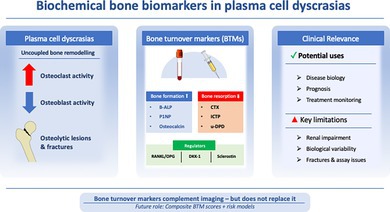

## INTRODUCTION

Multiple myeloma (MM) is the second most prevalent haematological malignancy in adults, characterised by the progressive accumulation of malignant plasma cells in the bone marrow.^1^ Despite substantial advances over the past two decades in combination treatment strategies and immunotherapies, which have significantly improved patient outcomes,^2^ a large proportion of patients already have established myeloma bone disease (MBD) at the time of diagnosis. Consequently, the management of MBD has become increasingly critical, both at initial presentation and throughout the disease course, affecting more than 90% of patients.^1^ Importantly, while bone‐targeted therapies such as bisphosphonates are effective in reducing the incidence of new skeletal‐related events (SREs), they are unable to reverse existing bone damage. Hence, there is clinical importance of early intervention and sustained prevention strategies to mitigate morbidity and mortality associated with skeletal complications.^3^

The histopathological hallmark of MBD is the progressive disruption of the balance between bone formation, driven by osteoblasts, and bone resorption, mediated by osteoclasts. Pathological plasma cells influence bone structure progressively both directly and through their interaction with the bone marrow microenvironment.^4^

Bone turnover markers (BTMs) are biochemical markers of bone physiology and pathology that are measured using non‐invasive tests, most commonly blood and urine assays. They are either directly produced by osteoblasts and osteoclasts or arise as by‐products of collagen synthesis and degradation.^5^ BTMs are classified as markers of bone formation or bone resorption, reflecting osteoblastic and osteoclastic activity, respectively, and ultimately bone homeostasis.^6^

Clinically, BTMs are utilised to predict bone loss and fracture risk as well as to assess treatment response in conditions affecting bone metabolism. Key biomarkers include markers of bone formation, such as procollagen type I N‐terminal propeptide (P1NP), and markers of bone resorption, such as C‐terminal telopeptide of type I collagen (CTX). These biomarkers are widely used in osteoporosis and MBD to evaluate skeletal health, monitor disease progression and assess response to bone‐targeted therapies. In MM, BTMs have enhanced our understanding of MBD pathogenesis and contributed to its clinical management, serving as both predictive markers of morbidity and prognostic indicators for the malignancy.^7^

Moreover, BTMs have demonstrated the impact of novel myeloma therapies on bone metabolism, particularly the strong antiresorptive effects of agents such as bortezomib.^8^ Additionally, immunomodulatory drugs (IMiDs), including lenalidomide and pomalidomide, have been shown to influence bone turnover by modulating osteoclast and osteoblast activity.^9^ As new treatments and combinations emerge for MBD, markers of bone formation could facilitate the monitoring of routinely used bone‐targeted therapies as well as novel anabolic agents.

In this review, we examine the literature on each BTM investigated across various plasma cell dyscrasias (PCDs), categorising their most common clinical applications and proposing directions for future research and potential clinical utility. Our objective is to provide a comprehensive review of the role of BTMs in PCDs, highlighting technical challenges, clinical limitations and potential future applications of these assays in the management of PCDs.

## BONE BIOMARKERS‐CLASSIFICATION AND CLINICAL RELEVANCE

Bone biomarkers are categorised into three main groups: bone formation markers, bone resorption markers and bone remodelling markers. Formation markers reflect osteoblastic activity and include proteins involved in bone matrix synthesis, while resorption markers indicate osteoclastic breakdown of bone collagen. Bone remodelling markers capture dynamic interactions between these two processes, providing a more comprehensive view of skeletal metabolism. Table 1 summarises the markers of bone formation, bone resorption and bone remodelling markers.

**TABLE 1:** The different bone biomarkers that could be used within plasma cell dyscrasias (PCDs). It highlights the advantages and disadvantages of each biomarker.

Biomarker	Classification	Mechanism	Relevance in MM	Relevance in MGUS and SMM	Advantages	Limitations
Bone‐specific alkaline phosphatase^10^	Bone formation	Hydrolyses pyrophosphate to promote bone mineralisation	Lower in MM due to suppressed osteoblast function.	No significant change in MGUS and SMM.	Minimal circadian variation. Routinely measured in clinical labs. Unaffected by food intake.	Lacks specificity for MM. Cross‐reacts with liver ALP isoform, reducing accuracy in patients with liver dysfunction.
Procollagen type I C‐terminal propeptide^11^	Bone formation	Released during type I collagen synthesis	Lower in MM due to supressed osteoblast function.	Lower in MGUS and SMM.	Reliable marker of collagen deposition. Unaffected by renal function.	Not specific to MM. Unable to distinguish between normal bone turnover and pathological bone loss.
Procollagen type I N‐terminal propeptide^12^	Bone formation	Cleaved from type I collagen during matrix formation	Declines in MM due to osteoblast suppression.	Limited change in MGUS and SMM.	Low biological variability. Stable in serum. Commonly used in bone turnover assessment.	Limited evidence in MM. Lacks validated cut‐offs for disease staging.
Osteocalcin^13^	Bone formation	Secreted by osteoblasts during matrix mineralisation	Decreased in MM due to reduced osteoblast activity.	No significant change in MGUS and SMM.	Correlates with bone formation rate. Useful for assessing treatment response.	Highly variable. Influenced by vitamin K status, limiting reliability in patients on anticoagulants. Requires specialised assays.
Periostin^14^	Bone formation	Stimulates osteoblasts via integrin receptors and Wnt‐β‐catenin pathway	Once disease progresses to MM, periostin levels stabilises. This correlates with early disease progression.	Increased in MGUS and SMM.	Associated with osteoblast activity. Potential biomarker for identifying skeletal metastases and bone fragility.	Lacks specificity for MM. Also elevated in lung, breast and gastrointestinal cancers. Not routinely measured in clinical settings.
Osteoprotegerin^15^	Bone remodelling regulator	Binds RANKL to inhibit osteoclast differentiation	Remains paradoxically high in MM despite osteolysis.	Increased in MGUS and SMM.	Reduces bone resorption. Potential therapeutic target in MM. Readily measurable in serum.	May not accurately reflect disease progression in MM. Lacks predictive power for bone damage.
Carboxy‐terminal pyridinoline cross‐linked telopeptide^16^	Bone resorption	Released from type I collagen breakdown by matrix metalloproteinase (MMP) activity	Elevated in MM and correlates with osteolytic disease.	No significant rise in MGUS or SMM.	Correlates with skeletal disease burden in MM. Unaffected by circadian variation.	Influenced by renal function, leading to potential overestimation in renal impairment. Less specific for osteoclast‐driven bone resorption than CTX.
Urinary N‐telopeptide of type I collagen^17^	Bone resorption	Excreted as degraded collagen crosslinks in urine	Increased in MM.	Slight increase in SMM. No significant change in MGUS.	Useful for monitoring response to bisphosphonate therapy. Readily measurable in urine samples.	Highly variable. Requires urine collection, making it less practical than serum‐based markers. Affected by hydration status.
Serum carboxy‐terminal collagen crosslinks^18^	Bone resorption	Released during osteoclastic degradation of type I collagen	Significantly elevated in MM.	Moderate increase in SMM. No major rise in MGUS.	Highly sensitive to changes in bone resorption. Routinely used in osteoporosis and MM.	Strong circadian variation. Requires fasting samples for reliable results. Rapidly decreases after bisphosphonate therapy, limiting long‐term monitoring value.
Urinary deoxypyridinoline^19^	Bone resorption	By‐product of collagen degradation, excreted in urine	Levels are normally higher in MM.	Levels can be normal or lower in SMM or MGUS.	Differentiates MGUS from MM. Used as an indicator of bone turnover.	Low sensitivity for early‐stage MM. Requires urine collection, which introduces variability. Not commonly used in clinical practice.
Cathepsin K^20^	Osteoclastic enzyme	Secreted by osteoclasts to degrade type I collagen	Increased in MM due to excessive bone resorption.	No major change in MGUS or SMM.	Key enzyme involved in osteoclast function. Targeted by investigational anti‐resorptive therapies.	Unstable at room temperature. Difficult to measure reliably in clinical settings.
Receptor activator of nuclear factor kappa‐B ligand^15^	Bone remodelling regulator	Stimulates osteoclast differentiation and function	Elevated in MM with RANKL/OPG ratios increasing as disease progresses.	No significant rise in MGUS or SMM.	Correlates with MM severity and the presence of osteolytic lesions. Serves as a therapeutic target for denosumab.	RANKL/OPG ratio is more informative than RANKL alone. Circulating levels may not fully reflect bone microenvironment dynamics.
Dickkopf‐1^21^	Bone remodelling regulator	Inhibits Wnt signalling, suppressing osteoblast differentiation	Highly elevated in MM.	Moderate increase in SMM. No significant change in MGUS.	Directly linked to MM‐induced osteolysis. Targeted in emerging MM therapies, such as anti‐DKK1 antibodies.	High inter‐patient variability. Lacks standardised clinical cut‐offs for routine use.
Sclerostin^22^	Bone remodelling regulator	Inhibits osteoblast differentiation and bone formation	Significantly elevated in MM.	Slight rise in SMM. No increase in MGUS.	Elevated in MM‐related osteolysis. Potential therapeutic target for anabolic bone therapies.	Affected by immobilisation, diabetes and metabolic conditions. Levels do not always correlate with MM disease activity.

Abbreviations: ALP, alkaline phosphatase; DKK1, Dickkopf‐related protein 1; MGUS, monoclonal gammopathy of undetermined significance; MM, multiple myeloma; OPG, osteoprotegerin; RANKL, receptor activator of nuclear factor kappa‐B ligand; SMM, smouldering MM, Wnt, wingless‐related integration site.

### Markers of bone formation

Markers of bone formation primarily reflect osteoblast activity and matrix deposition. Alkaline phosphatase (ALP), particularly its bone‐specific isoform (bone‐specific alkaline phosphatase, B‐ALP), is instrumental in osteoid formation and mineralisation by hydrolysing pyrophosphate, an inhibitor of mineral deposition. Elevated B‐ALP levels are generally indicative of increased bone turnover, and in pathological states such as osteoporosis and myeloma, higher levels have been associated with an increased risk of fracture, reflecting uncoupled or ineffective bone remodelling rather than protective bone formation.^10^ Conversely, reductions in B‐ALP following antiresorptive or anti‐myeloma therapy are interpreted as evidence of suppressed pathological bone turnover, which is associated with reduced fracture risk.

Similarly, procollagen type I C‐terminal propeptide and P1NP serve as markers of collagen deposition during bone matrix formation, making them reliable indicators of osteoblastic activity across various physiological and pathological conditions.^11^ Another key protein biomarker, osteocalcin, is a calcium‐binding protein synthesised by osteoblasts, odontoblasts and hypertrophic chondrocytes. It is particularly elevated in metabolic bone diseases with increased osteoid formation, such as osteoporosis and osteomalacia, as well as in patients with fractures and bone metastases. Osteocalcin levels have also been used to monitor treatment response in osteoporosis and hypercalcaemia.^14^ Additionally, periostin, a structural protein found in collagen‐rich connective tissues, has been implicated in tumour metastasis to the bone, particularly in lung and breast cancers. Beyond its structural role, periostin acts as a signalling molecule, stimulating osteoblast function and bone formation through integrin receptors and the Wnt‐β‐catenin pathway.^14^

### Markers of bone resorption

In contrast, markers of bone resorption provide insights into osteoclast activity and collagen degradation. Telopeptides released during type I collagen breakdown, such as carboxy‐terminal pyridinoline (PYD) cross‐linked telopeptide (ICTP),^16^ urine N‐telopeptide of type I collagen (NTX) and CTX,^18^, ^23^ are widely used to assess bone resorption and monitor conditions such as osteoporosis.^24^ Urinary deoxypyridinoline (u‐DPD), a collagen crosslink product excreted during bone degradation, has been proposed as a marker of bone turnover, particularly in breast cancer.^19^

Osteoclastic enzymes and osteocyte‐derived factors further contribute to bone resorption. Cathepsin K, a proteolytic enzyme secreted by osteoclasts, plays a crucial role in collagen degradation and bone remodelling.^6^ Its' levels have been shown to decrease in postmenopausal women receiving oral alendronate therapy,^20^ although a specific enzyme‐linked immunosorbent assays (ELISA) assay for its active form failed to detect significant changes in osteoporotic women on antiresorptive therapy.^25^

The cytokines osteoprotegerin (OPG) and receptor activator of nuclear factor kappa‐B ligand (RANKL) are regulators of osteoclast differentiation, survival and activation.^15^ Importantly, osteoclast activity is determined by the balance between RANKL and OPG rather than their absolute concentrations, with the RANKL:OPG ratio providing a more informative reflection of net osteoclastogenic signalling. Acting as a decoy receptor for RANKL, OPG inhibits osteoclastogenesis, thereby reducing bone resorption and the development of osteolytic lesions.^26^

### Markers of bone remodelling

Beyond the factors listed above, several other regulatory proteins modulate bone remodelling, including Dickkopf‐related protein 1 (DKK1)^27^ and sclerostin (SCL).^28^ DKK1 is a key inhibitor of the Wnt signalling pathway, which is essential for osteoblast differentiation and activity. SCL, a secreted glycoprotein produced by osteocytes, exerts a dual effect by suppressing osteoblast differentiation via the Wnt pathway while promoting osteoclastic bone resorption.^6^, ^28^ However, it is important to note that serum SCL levels may not directly reflect local bone activity. While systemic levels have been shown to increase with factors such as age, direct measurements in bone tissue indicate no proportional rise in osteocyte‐derived SCL expression.^29^ This suggests that circulating SCL is regulated by additional systemic factors, such as reduced renal clearance or altered protein turnover, rather than directly reflecting bone metabolic activity.

### Clinical application of bone biomarkers in MM

In MM, the relevance of bone biomarkers differs from that in other disorders of bone remodelling. While markers of bone formation such as B‐ALP have shown limited clinical relevance, they may still be useful in assessing response to anti‐myeloma therapies.^30^ In contrast, bone resorption markers have demonstrated greater utility in understanding the impact of malignant plasma cells on bone metabolism. Myeloma cells actively disrupt bone homeostasis by interfering with key pathways, such as the sRANKL (soluble receptor activator of nuclear factor kappa‐beta ligand)/OPG axis and DKK1 signalling, leading to excessive bone resorption and the development of osteolytic lesions.^26^

In clinical practice, several biomarkers have been explored within the management of MM. Serum CTX has been investigated for its role in the early diagnosis of MBD and in determining the need for bisphosphonate therapy.^31^, ^32^ u‐DPD and SCL have been associated with disease prognosis,^33^, ^34^ while the RANKL/OPG ratio has emerged as a potential therapeutic target in anti‐myeloma treatment strategies.^35^

## APPLICATIONS AND LIMITATIONS OF MEASURING BONE BIOMARKERS IN PCDs

The use of bone biomarkers in diagnosing and monitoring bone disease in PCDs requires careful consideration of laboratory methods, reproducibility, availability, costs, sensitivity to renal function, fasting status and circadian variability. Table 1 summarises the advantages and disadvantages of each bone biomarker that could be utilised for the management of PCDs.

Among bone formation markers, B‐ALP and P1NP have demonstrated minimal circadian fluctuation (*p* > 0.05)^12^ and limited dietary influence,^36^ making them suitable for routine clinical use. B‐ALP, constituting approximately 50% of circulating ALP,^37^ is commonly measured using automated immunoassays.^12^ However, cross‐reactivity with the liver isoform (~15%) remains a limitation, particularly in patients with hepatic dysfunction. Serum P1NP, which exhibits low biological variation (co‐efficient of variability [CV] <10%) and interassay variability <5%, is widely used for assessing bone turnover.^38^

Osteocalcin, another bone formation marker, exhibits significant heterogeneity due to its rapid metabolism within immunochemical and chromatographic studies in normal individuals and in patients with osteoporosis, chronic renal failure and Paget's disease.^39^ Immunoassays measuring intact osteocalcin (amino acids 1–49) and the N‐terminal/mid‐region fragment (amino acids 1–43) show variations in analytical stability, with the latter proving more consistent.^40^ Serum osteocalcin concentrations are significantly influenced by vitamin K status (*p* < 0.01),^41^ necessitating caution in patients receiving vitamin K antagonists. Periostin, an emerging marker, is independent of bone mineral density (BMD) and traditional bone markers, but its lack of skeletal specificity (elevated in aortic, gastrointestinal and uterine tissues) limits its diagnostic utility.^42^

Among bone resorption markers, type I collagen telopeptides (CTX and ICTP) exhibit significant circadian variability. Serum ICTP levels peak at night (~20% higher than afternoon values, *p* = 0.003) and fluctuate in parallel with insulin‐like growth factor I levels (*p* < 0.01), necessitating fasting sample collection.^43^ CTX, a widely used marker of bone degradation, shows a 40% circadian variation (*p* < 0.001), which is reduced by 75% in fasting individuals (*p* < 0.01). Long‐term sample stability is an advantage of CTX, with concentrations remaining stable in frozen serum and plasma for up to 3 years.^18^

u‐DPD, another marker of bone degradation, does not require fasting and morning urine samples are preferred for standardisation. It is measured by enzyme immunoassay on microtitre plates.^19^ The u‐DPD enzyme immunoassay exhibits intra‐assay and interassay CVs of <10% and <15%, respectively, with minimal cross‐reactivity (<1%) with PYD.^44^

Dickkopf‐1 (DKK‐1), a Wnt signalling antagonist, plays a key role in inhibiting osteoblast differentiation and function. Elevated serum DKK‐1 levels have been reported not only in patients with MM (*p* < 0.001) but also in monoclonal gammopathy of undetermined significance (MGUS), where levels correlate with increased osteolytic activity and impaired bone formation.^45^ These findings support a role for early disruption of Wnt signalling in the pathogenesis of myeloma‐related bone disease.

Current assays for DKK‐1 rely on manual ELISA, which show moderate reproducibility (coefficient of variation <15%).^21^ Additionally, serum DKK‐1 levels fluctuate according to disease response to anti‐myeloma therapies, potentially confounding its role as a stable biomarker for longitudinal monitoring.^46^

Other biomarkers, including cathepsin K, RANKL, OPG and SCL, face similar challenges. Cathepsin K, a specific osteoclast marker, is unstable at room temperature, limiting its widespread application. RANKL and OPG, key regulators of bone metabolism, exhibit low circulating concentrations and moderate assay reproducibility (CV ~15%), making them difficult to quantify reliably.^6^ RANKL serum measurements may not accurately reflect bone microenvironment dynamics.^47^ SCL, measured via immunoassay, and DKK‐1 are biochemical markers that are significantly influenced by physical immobilisation and are both raised in metabolic conditions such as diabetes (*p* < 0.001), limiting its specificity in PCDs.^48^, ^49^, ^50^, ^51^

## EFFECTS OF FRACTURE ON BONE TURNOVER MARKERS

One major limitation of bone biomarkers in clinical monitoring is their prolonged elevation following fractures, which restricts their utility in tracking disease progression in PCDs. Bone resorption markers typically increase within the first 4 weeks post‐fracture, followed by a rise in bone formation markers. This elevation, estimated at 20%–50%, may persist for up to 6 months.^5^ In postmenopausal women with distal forearm fractures, both resorption and formation markers remain elevated for up to 52 weeks, reflecting different stages of bone healing and mineralisation.^52^ Similarly, following a tibial fracture, CTX levels rise within 3 days, peaking in the subsequent 2 weeks, whereas bone formation markers such as B‐ALP and P1NP peak at 12 and 24 weeks respectively.^53^ Furthermore, the considerable interindividual variability in bone turnover responses makes it impractical to apply a standardised fracture adjustment, further limiting the clinical utility of these biomarkers.

## USE OF BONE BIOMARKERS IN RENAL IMPAIRMENT

Renal dysfunction, a frequent complication in MM, significantly affects bone biomarker levels, as most markers are cleared by the kidneys. Among commonly used markers, only tartrate‐resistant acid phosphatase‐5b and B‐ALP are not renally excreted.^54^, ^55^ As B‐ALP is not eliminated via the kidneys, it is the preferred biomarker for assessing bone disease in chronic kidney disease (CKD), a frequent condition associated with myeloma.^56^ Conversely, P1NP, though primarily metabolised by the liver, accumulates in CKD, with intact P1NP assays proving more reliable than total P1NP assays.^57^

Urinary markers such as PYD and u‐DPD show poor reliability in renal impairment.^58^ In MM patients with renal dysfunction, u‐DPD did not correlate with lytic bone disease (*p* = 0.081), but remained an independent prognostic factor, together with CRP (C‐reactive protein), for survival (*p* < 0.001).^33^ Similarly, CTX exhibits high variability in CKD and haemodialysis patients, with values correlating significantly with glomerular function (*p* < 0.001).^11^ ICTP, which is also renally excreted, loses prognostic value in CKD, whereas serum NTX is more specific for bone resorption in this subset, as it is less affected by renal clearance compared to urinary NTX (u‐NTX), which depends on kidney excretion.^24^

Serum OPG and RANKL are increased in pre‐dialysis and dialysis patients, but soluble RANKL remains stable across CKD stages.^59^, ^60^ The RANKL/OPG ratio is correlated with BMD in CKD patients, supporting its potential use in this population.^61^

Markers such as osteocalcin exhibit significant variability in CKD due to differences in vitamin K status, affecting carboxylation and requiring specialised assays for accurate measurement.^41^ Periostin levels correlate with renal function decline in animal models, with periostin inhibition protecting against nephropathy and fibrosis, suggesting a potential role in CKD‐associated bone disease.^62^

Serum SCL increases in advanced CKD and correlates with parathyroid hormone (PTH), phosphate and vitamin D levels, whereas DKK‐1 levels are lower in CKD patients than in controls and do not correlate with mineral metabolism markers.^63^

## MARKERS OF BONE METABOLISM IN PCDs

### Use of bone biomarkers in classification of gammopathies and disease progression

**FIGURE 1:**
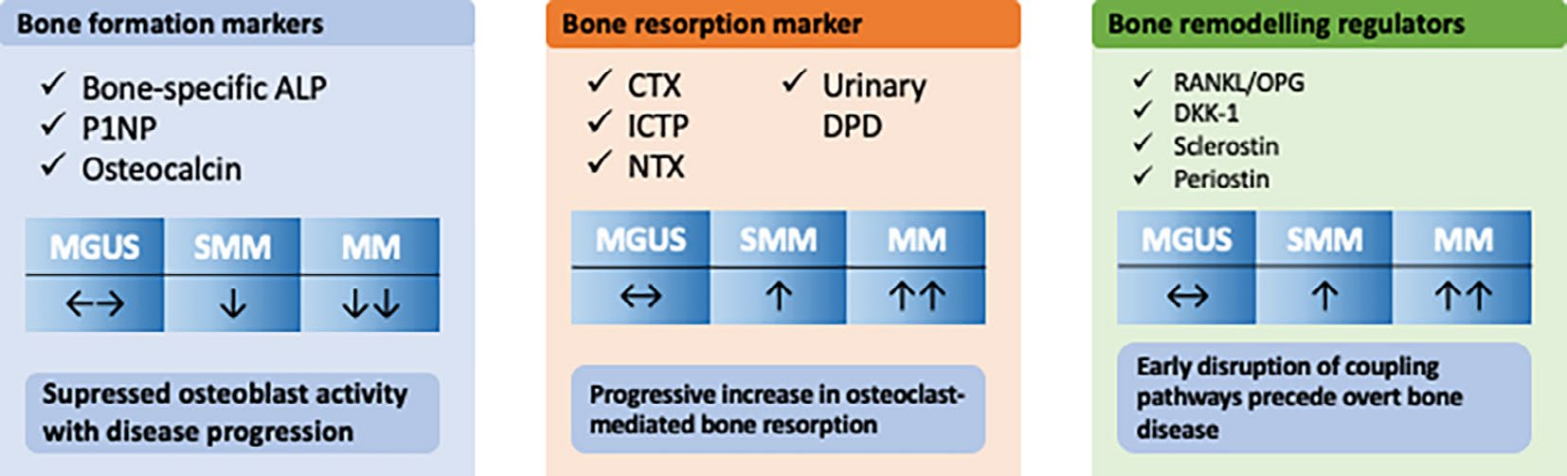
Classification of bone turnover markers and their association with disease stage in plasma cell dyscrasias. Schematic overview of commonly used bone formation markers (bone‐specific alkaline phosphatase [B‐ALP], procollagen type I N‐terminal propeptide [P1NP] and osteocalcin), bone resorption markers (C‐terminal telopeptide of type I collagen [CTX], N‐terminal telopeptide [NTX] and urinary deoxypyridinoline [DPD]), and regulators of bone remodelling (RANKL/osteoprotegerin ratio, DKK‐1, sclerostin, periostin). Relative changes in marker levels across monoclonal gammopathy of undetermined significance (MGUS), smouldering multiple myeloma (SMM) and multiple myeloma (MM) are indicated (↔, no significant change; ↓/↑, moderate decrease/increase; ↓↓/↑↑, substantial decrease/increase).

Bone biomarkers have been primarily studied in distinguishing symptomatic MM from its asymptomatic precursors, MGUS and smouldering MM (SMM), as well as from healthy controls. Some studies have also explored biomarker differences between active myeloma and post‐treatment states. A summary of these biomarkers and their association with disease states is provided in Figure 1.

Among bone formation markers, only B‐ALP and periostin appear useful in disease subclassification. B‐ALP levels are lower in active MM than in MGUS or healthy controls, reflecting reduced osteoblast activity.^64^, ^65^ Periostin is among the extracellular matrix proteins that are progressively upregulated in MGUS and MM, suggesting a potential role in disease pathogenesis.^66^ In newly diagnosed MM (NDMM), serum periostin is significantly elevated (almost fourfold higher) compared to healthy controls, SMM and MGUS but does not significantly differ between NDMM and relapsed MM patients.^67^

Other bone formation markers, such as P1NP and osteocalcin, lack clear discriminative power. P1NP levels do not significantly differ between NDMM, MGUS or SMM nor across Durie‐Salmon stages in symptomatic MM.^68^ Osteocalcin levels vary inconsistently in NDMM populations compared to controls.^69^

Bone resorption markers demonstrate better diagnostic accuracy. ICTP and CTX are significantly elevated in NDMM compared to MGUS and healthy controls^70^ and u‐DPD excretion is a key differentiator between MGUS and MM (*p* = 0.0058).^71^ However, while MGUS detection specificity is 93%, sensitivity for stage I MM is only 58%, limiting early‐stage MM detection.^71^ Despite RANKL/OPG pathway involvement in myeloma pathogenesis, serum OPG levels are paradoxically higher in both MGUS and MM than in controls, likely due to alternative sources such as the stromal cells.^72^

In MGUS patients without osteolytic lesions, increased osteoclast activity precedes detectable bone damage. CTX and P1NP levels are significantly higher in progressing MGUS cases than in stable ones.^73^ Elevated RANKL and RANKL/OPG ratios correlate with higher risk MGUS and SMM, supporting their role in predicting disease progression.^73^, ^74^, ^75^

With regard to prognosis of MM, ICTP is the only bone marker consistently demonstrating prognostic value for survival in MM in a non‐clinical setting.^16^, ^76^ Additionally, RANKL upregulation is linked to disease progression, with increased levels correlating with anaemia, immunosuppression and tumour burden.

A prospective study in NDMM patients showed that CTX‐I increases significantly (~41%) 1 month before progression detection, making it a potential early marker of disease relapse.^31^ B‐ALP also increased by 17% at the first sign of progressive disease, suggesting bone formation‐resorption imbalance as an early marker of disease progression.^31^ Periostin levels correlate with poor prognostic factors such as β2‐microglobulin, lactate dehydrogenase (LDH) and high International Staging System (ISS) stage, indicating its potential as a disease progression biomarker.^77^

### Myeloma bone disease

Although not included in routine guidelines, BTMs have been investigated for diagnosing and monitoring MBD. B‐ALP correlates with bone involvement severity and disease progression but requires further validation for clinical use.^31^ u‐DPD correlates with myeloma‐related bone disease, with significantly higher levels in patients with both limited and extensive skeletal lesions on X‐ray.^70^

Among bone resorption markers, CTX and ICTP correlate with skeletal disease burden and fracture risk. ICTP is significantly elevated in myeloma patients with magnetic resonance imaging (MRI)‐detected bone lesions despite a normal skeletal survey, suggesting its potential in identifying early bone disease.^76^ u‐NTX correlates with bone involvement on Tc‐99m‐MIBI scintigraphy and plasma cell infiltration in the bone marrow.^17^

Serum CTX is significantly higher in patients with osteolytic lesions and pathological fractures, whether detected by X‐ray,^78^ computed tomography (CT), MRI or positron emission tomography‐computed tomography (PET‐CT).^79^ In patients with negative skeletal surveys, elevated CTX has a 91.3% positive predictive value for bone disease on MRI.^78^ Similarly, CTX elevation correlates with bone marrow plasma cell infiltration and lytic lesions, supporting its role in detecting subclinical bone involvement.^80^

In addition, OPG alone has been identified as a marker of bone disease in MM patients but does not appear to have additional prognostic value in myeloma progression.^81^ Finally, soluble RANKL and DKK‐1 correlate with radiographically evident bone disease, supporting their role in myeloma‐related skeletal pathology.^82^

### Assessment of response to anti‐myeloma treatment

While MBD has been extensively reviewed in the context of evolving systemic therapies, including a recent review,^83^ variability in the direction, magnitude and clinical interpretability of therapy‐specific changes in individual markers remains an area of ongoing uncertainty.

#### Proteasome inhibitors‐Bortezomib and carfilzomib

Proteasome inhibitors influence bone metabolism in MM, with bortezomib shown to restore osteoblast differentiation via vitamin D pathways^84^ and enhance bone formation biomarkers, likely mediated via modulation of osteoblast regulatory pathways and normalisation of Wnt inhibitors.^31^, ^85^, ^86^ In clinical studies, B‐ALP and P1NP levels rise significantly within three treatment cycles in responders to bortezomib‐containing regimens (e.g. UARK 2001‐37, SUMMIT [Study of Uncontrolled Multiple Myeloma Managed with Proteasome Inhibition Therapy] and APEX [Assessment of Proteasome Inhibition for Extending Remissions]), suggesting an association with bone formation and osteoblast activity.^31^, ^87^, ^88^

However, its clinical significance remains uncertain, with variable results on osteocalcin and differential responses depending on concomitant therapies and bisphosphonate use.^85^, ^86^, ^88^, ^89^ Bortezomib also reduces circulating inhibitors of bone formation, such as DKK‐1, further supporting its osteoblast‐stimulatory potential.^90^

Carfilzomib also increases B‐ALP, supporting its potential bone‐anabolic effects.^91^ This was later confirmed by micro‐CT studies, which showed an increase in bone architectural parameters, such as bone volume to total volume fraction and trabecular thickness in 80% and 70%, respectively, of responders.^91^

#### Immunomodulatory drugs

IMiDs such as lenalidomide and thalidomide have variable effects on BTMs. Preclinical data suggest that they may inhibit osteoclastogenesis via suppression of RANKL, upregulation of OPG and downregulation of cathepsin K.^92^, ^93^ However, clinical validation of BTMs as markers of response to lenalidomide remains limited, with the most consistent finding being modest CTX reductions in responders without correlated structural changes.^79^

#### Daratumumab

Recent prospective data from the phase‐2 REBUILD trial (NCT03475628) demonstrate that daratumumab, an anti‐CD38 (cluster of differentiation 38) monoclonal antibody, exerts favourable effects on bone turnover in relapsed/refractory MM.^94^ Osteocalcin, B‐ALP and P1NP increased from baseline over 4 months of therapy, while levels of DKK‐1 and other inhibitory factors decreased, indicating enhanced bone formation and reduced osteoblast inhibition. Changes in resorption markers (CTX and tartrate‐resistant acid phosphatase isoform 5b [TRACP‐5b]) showed non‐significant reductions, suggesting a predominant effect on formation pathways. Hence, daratumumab may positively modulate bone metabolism beyond its anti‐myeloma effects.

#### Emerging cellular therapies

Preliminary retrospective analyses suggest that B‐cell maturation antigen (BCMA)‐targeted chimeric antigen receptor T‐cell (CAR‐T) therapies may also influence BTMs, with reported reductions in bone resorption markers and increases in osteocalcin in responding patients, though data remain limited and further investigation is needed to define these effects.^95^, ^96^

### Assessment of response to bone‐targeted agents

Bisphosphonate therapy significantly impacts BTMs, particularly CTX, P1NP and NTX. Zoledronic acid (ZA) significantly reduces CTX (*p* < 0.0001) and B‐ALP (*p* = 0.0042), with effects influenced by cumulative dose and infusion frequency.^97^ However, higher cumulative ZA infusions have been associated with declines in renal function, though renal toxicity is often multifactorial and may also occur idiosyncratically.^97^

The Z‐MARK trial (Zolendronic acid‐Bone MARK‐er‐Directed dosing) demonstrated that u‐NTX monitoring over 4 years allowed reduced ZA dosing while maintaining low SRE rates.^98^ However, low baseline NTX levels limit its predictive utility. However, when used to address the dose adjustment of ZA on a 4‐year follow‐up, monitoring of u‐NTX levels helped maintaining a low SRE rate even in patients receiving ZA less frequently.^98^

In a cohort of 178 Chinese MM patients receiving anti‐myeloma therapy plus bisphosphonates, CTX/P1NP ratios significantly decreased, independent of disease burden at diagnosis or the International Myeloma Working Group (IMWG) response criteria.^99^ Hence, bisphosphonate effects on bone turnover are sustained regardless of tumour response, further supporting the role of CTX and P1NP in long‐term skeletal monitoring.

### Novel bone biomarkers

Over the last few years, several novel bone biomarkers have been reported, each adding a different window on the biology of MBD. Secreted frizzled‐related protein 3 is the only Wnt pathway inhibitor shown to be consistently overexpressed in myeloma‐associated lytic bone disease. Its expression parallels bone resorption activity, which makes it an attractive candidate biomarker and a potential therapeutic target in MBD.^100^

C‐type natriuretic peptide is a regulator of endochondral growth and circulates mainly as its amino‐terminal propeptide, NT‐proCNP (N‐terminal pro‐C‐type‐natriuretic peptide). In myeloma, serum NT‐proCNP tracks with formation markers, although interpretation is limited by age, renal impairment and exposure to steroids, which all influence concentrations.^101^

Chitinase‐3‐like protein 1, also known as YKL‐40, participates in extracellular matrix remodelling and inflammation and has functional links to osteoclast biology. In cohorts treated with anti‐myeloma therapy and pamidronate, higher serum YKL‐40 was associated with earlier bone disease progression and poorer survival, although its independent prognostic value still needs confirmation.^102^

Growth differentiation factor 15 (GDF‐15) is overexpressed by bone marrow stromal cells in myeloma and contributes to plasma cell growth and drug resistance. Circulating GDF‐15 is higher in patients with extensive osteolytic lesions, consistent with experimental data showing promotion of osteoclastogenesis and inhibition of osteoblast differentiation.^67^

Semaphorin 3A, an antiangiogenic factor, has been implicated in the transition from MGUS to MM,^103^, ^104^ while Semaphorin 4D, via Plexin‐B1, shifts the balance between osteoclasts and osteoblasts by suppressing osteoblast differentiation and altering cell motility. In newly diagnosed disease, raised bone marrow and serum Semaphorin 4D associate with osteolytic lesions, higher ISS stage and diffuse MRI marrow infiltration, linking it to bone burden and overall disease activity.^105^

Tumour necrosis factor superfamily member 14 (TNFSF14), despite being known to be involved in MM for almost a decade, is now re‐emerging as a regulator of bone remodelling with biomarker potential. Early work showed that TNFSF14 is overproduced by monocytes, CD8 T cells and neutrophils in patients with MBD and that it drives osteoclast formation while suppressing osteoblast differentiation.^106^ Neutralising TNFSF14 in patient‐derived cultures reduced osteoclastogenesis, and its effect appeared additive to RANKL. More recently, clinical studies reported persistent TNFSF14 overexpression in circulating monocytes among patients who continued to have active bone disease despite anti‐myeloma treatment, supporting TNFSF14 as a readout of ongoing skeletal injury rather than tumour burden.^107^ However, it is worth noting that assays are still research grade, typically ELISA or flow cytometry, and there are no validated clinical cut‐offs. Hence, it is still mainly utilised in research settings.

Circulating and exosomal microRNAs are also beginning to show potential as bone‐linked biomarkers that capture communication between myeloma cells, stromal cells and bone. Panels enriched for bone relevant species, including miR‐21, miR‐16 and the miR‐29 family, have been associated with osteolytic burden, risk of progression and early treatment effects.^108^, ^109^, ^110^ Pre‐analytical handling and platform variability remain the main barriers to clinical use, so at present, these signatures are best considered research tools that can enrich risk stratification across MGUS, SMM and active myeloma, highlight high resorption biology when imaging is inconclusive and serve as exploratory end‐points in prospective studies with standardised sampling and analysis plans.

## CONCLUSION AND FUTURE DIRECTIONS

BTMs offer valuable insights into bone metabolism in PCDs, reflecting changes in bone remodelling associated with MM and its precursor conditions. Their use has been explored in various clinical settings, including the detection of early bone disease in MGUS and SMM, monitoring disease progression and assessing response to bone‐targeted and anti‐myeloma therapies. Despite their potential, several limitations hinder routine clinical implementation. High intraindividual variability, along with the absence of osteocyte‐specific markers and differences in assay techniques and cut‐off values, limits their standardisation. Consequently, current IMWG, European Society of Medical Oncology (ESMO) and National Comprehensive Cancer Network (NCCN) guidelines do not include BTMs in routine myeloma diagnostics, though the 2010 IMWG report proposed u‐NTX, serum CTX and ICTP for selective use in clinical monitoring.^75^, ^111^, ^112^, ^113^, ^114^

Imaging remains the cornerstone for diagnosing and monitoring MBD, with the 2014 IMWG criteria recognising osteolytic lesions on CT and PET‐CT as definitive markers, independent of conventional radiographs.^113^, ^114^ While whole‐body low‐dose CT is now recommended, its widespread adoption is hindered by the need for standardisation in image acquisition, interpretation and reporting as well as financial and logistical constraints in many healthcare systems. Within this context, BTMs represent a potential adjunctive tool rather than a replacement for imaging, offering real‐time, cost‐effective insights into dynamic changes in bone metabolism before radiological changes occur. However, imaging remains essential for defining the extent of bone disease at diagnosis and for reassessment when clinically indicated, such as in the presence of new or worsening bone pain.

As novel anti‐myeloma therapies achieve deeper remissions and prolonged disease control, the importance of bone anabolism is increasing. Wnt pathway inhibitors such as DKK‐1 and SCL suppress osteoblast function, highlighting the need to evaluate bone formation alongside resorption. Monoclonal antibodies targeting these pathways are under investigation, including romosozumab against SCL and anti‐DKK‐1 antibodies, as potential osteoanabolic strategies in plasma cell disorders.^115^ Early prospective data with romosozumab demonstrate improvements in BMD and bone turnover markers without evidence of myeloma progression. While it remains uncertain whether individual BTMs can reliably distinguish between true bone formation and suppression of bone resorption in patients receiving bisphosphonates, denosumab or emerging osteoanabolic agents, this limitation highlights the need for integrated approaches to biochemical interpretation.

**FIGURE 2:**
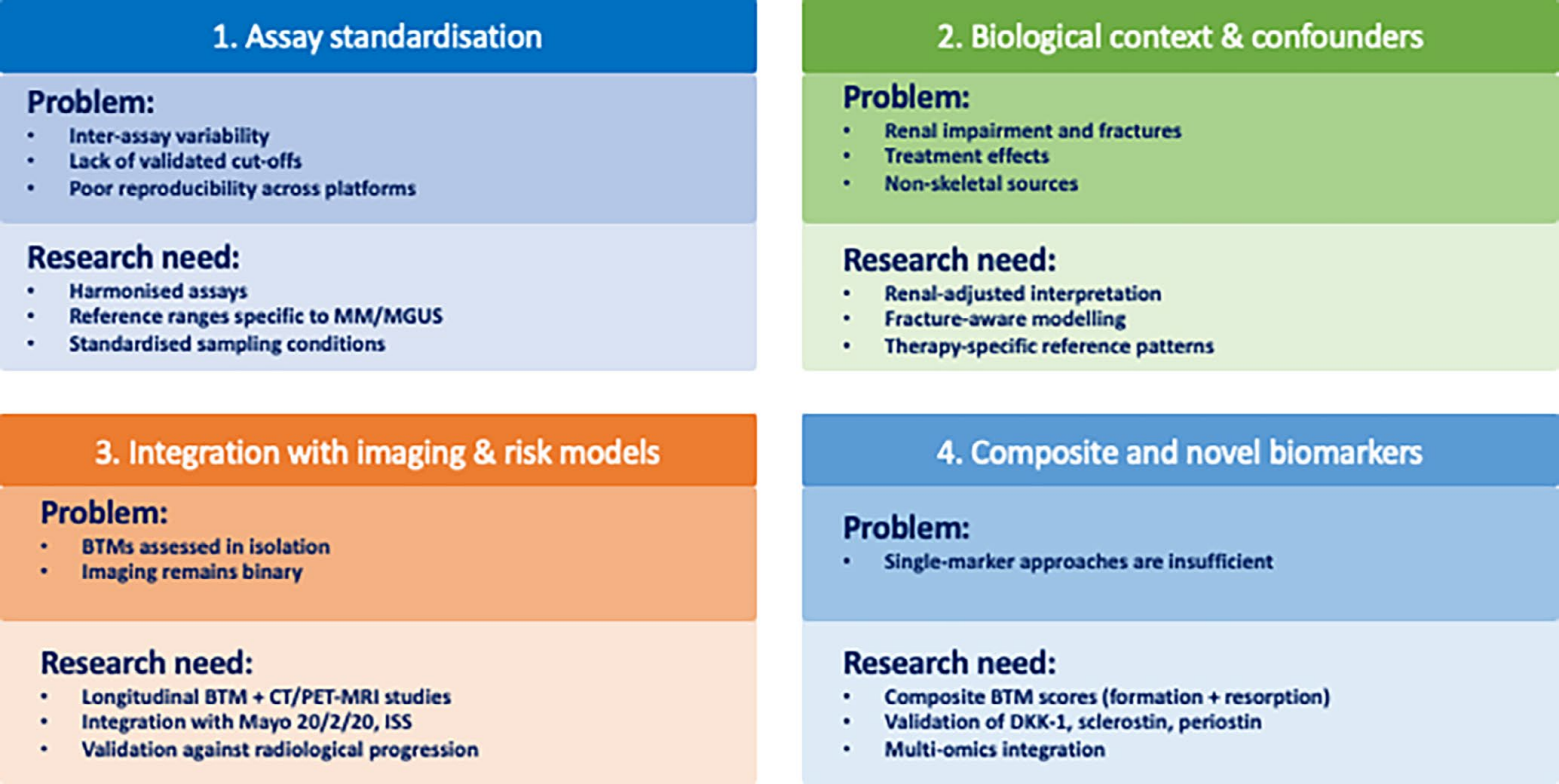
Future research priorities for bone turnover markers in plasma cell dyscrasias. Schematic summary of key methodological and translational challenges limiting the clinical application of bone turnover markers (BTMs).

Future research should focus on validating cut‐off values, improving assay reproducibility and establishing standardised interpretation criteria (summarised in Figure 2). The development of a composite BTM score, incorporating both bone formation and resorption markers, is likely to provide a more robust assessment of skeletal remodelling and may offer improved risk stratification for myeloma‐related bone disease compared to individual markers alone.

An important area of future investigation is the role of BTMs in predicting the progression from MGUS and SMM to symptomatic disease. Current risk models, such as the Mayo 20/2/20 system, rely on clinical and genetic parameters but do not integrate bone turnover dynamics.^116^ Longitudinal studies incorporating BTMs alongside traditional prognostic factors may refine risk stratification, allowing for earlier therapeutic intervention in high‐risk patients. Additionally, BTMs may facilitate a more personalised approach to bone‐targeted therapy by helping to determine the optimal duration and dosing of bisphosphonate or denosumab treatment. Their ability to predict early SREs, such as vertebral fractures or osteolytic progression before radiological detection, warrants further exploration. A deeper understanding of dynamic BTM changes during anti‐resorptive therapy could refine decision‐making regarding treatment escalation or de‐escalation.

The integration of emerging bone biomarkers, including periostin, GDF‐15, DKK‐1 and SCL, into clinical research may further enhance our understanding of MBD. Investigating their predictive value for bone disease severity, treatment response and disease progression could open new avenues for precision medicine approaches in myeloma. Advances in multi‐omics research, incorporating genomic, proteomic and metabolomic profiling, may offer novel insights into individualised risk assessment and therapeutic targeting. Additionally, the correlation between specific BTMs and radiological findings remains an area of active investigation. Determining whether BTM thresholds align with radiological progression could facilitate earlier intervention, potentially preventing irreversible bone damage.

As survival rates in myeloma improve, the long‐term management of bone health becomes increasingly important. Many myeloma survivors, particularly those receiving long‐term maintenance therapy with lenalidomide or other agents, face a persistent risk of bone fragility and fractures.^117^ Evaluating the role of BTMs in post‐treatment bone health monitoring may help optimise follow‐up strategies to reduce fracture risk, such as the timing and class of osteoporosis therapy to use in primary and secondary fracture prevention settings, such as fracture liaison services. Additionally, the impact of hormonal influences, PTH and vitamin D status, renal status and novel bone microarchitecture assessment tools, such as trabecular bone score and high‐resolution peripheral quantitative CT, could further refine long‐term skeletal monitoring strategies.

Overall, future research should focus on standardising their measurement, validating their predictive value and integrating them with imaging and precision medicine approaches to optimise bone disease management in myeloma and its precursor states. Addressing these challenges could establish BTMs as a key tool in personalised bone disease management in PCDs, improving clinical outcomes and long‐term survivorship strategies.

## AUTHOR CONTRIBUTIONS

GN and KR conceived the study and developed the original idea. GN and RV drafted the initial versions of the manuscript. KR reviewed the draft, contributed to manuscript development following the initial draft and provided administrative support. CME reviewed the manuscript and provided critical feedback. All authors contributed to the final version of the manuscript and approved it for submission.

## FUNDING INFORMATION

GN received research funding from Amgen. No other specific funding was received for the preparation of this review.

## CONFLICT OF INTEREST STATEMENT

GN reports research funding from Amgen. RV declares no conflicts of interest. CME declares no conflicts of interest. MKJ receives paid consultancy or sponsorship for external talks from Amgen, UCB, Sanofi and Kyowa Kirin. KR has received advisory fees, honoraria and research funding from AbbVie, Amgen, Bristol Myers Squibb, GSK, Janssen, Sanofi and Takeda.

## Data Availability

Data sharing not applicable to this article as no datasets were generated or analysed during the current study.
